# Identification of a blockade epitope of human norovirus GII.17

**DOI:** 10.1080/22221751.2021.1925162

**Published:** 2021-05-18

**Authors:** Yufang Yi, Xiaoli Wang, Shuxia Wang, Pei Xiong, Qingwei Liu, Chao Zhang, Feifei Yin, Zhong Huang

**Affiliations:** a Key Laboratory of Tropical Translational Medicine of Ministry of Education, Hainan Medical University, Haikou, People’s Republic of China; b CAS Key Laboratory of Molecular Virology & Immunology, Institut Pasteur of Shanghai, Center for Biosafety Mega-Science, Chinese Academy of Sciences, University of Chinese Academy of Sciences, Shanghai, People’s Republic of China; c Hainan Medical University – The University of Hong Kong Joint Laboratory of Tropical Infectious Diseases, Hainan Medical University, Haikou, People’s Republic of China

**Keywords:** Norovirus, GII.17 genotype, monoclonal antibody, epitope, virus-like particle, histo-blood group antigens

## Abstract

Human noroviruses are the dominant causative agent of acute viral gastroenteritis worldwide. During the winter of 2014–2015, genotype GII.17 cluster IIIb strains emerged as the leading cause of norovirus infection in Asia and later spread to other parts of the world. It is speculated that mutation at blockade epitopes may have resulted in virus escape from herd immunity, leading to the emergence of GII.17 cluster IIIb variants. Here, we identify a GII.17 cluster IIIb-specific blockade epitope by monoclonal antibody (mAb)-based epitope mapping. Four mAbs (designated as M1 to M4) were generated from mice immunized with virus-like particle (VLP) of a GII.17 cluster IIIb strain. Among them, M1 and M3 reacted specifically with the cluster IIIb VLP but not with the VLPs from clusters II or IIIa. Moreover, M1 and M3 dose-dependently blocked cluster IIIb VLP binding with its ligand, histo-blood group antigens (HBGAs). Epitope mapping revealed that M1 and M3 recognized the same highly exposed epitope consisting of residues 293–296 and 299 in the capsid protein VP1. Sequence alignment showed that the M1/M3 epitope sequence is highly variable among different GII.17 clusters whereas it is identical for cluster IIIIb strains. These data define a dominant blockade epitope of GII.17 norovirus and provide evidence that blockade epitope evolution contributes to the emergence of GII.17 cluster IIIb strains.

## Introduction

Human noroviruses, which belong to the *Norovirus* genus of the *Caliciviridae* family, are the dominant cause of acute viral gastroenteritis [1]. The viruses possess a single stranded, positive-sense RNA genome of 7.5–7.7 kb. The RNA genome contains three open reading frames (ORFs 1–3) encoding either nonstructural or structural proteins [2]. Specifically, ORF1 encodes nonstructural proteins required for virus replication while ORF2 and ORF3 encode the major capsid protein VP1 and a minor capsid protein VP2, respectively [3]. VP1 protein can be divided into a shell (S) domain and a protruding (P) domain, and the latter can be further subdivided into P1 and P2 subdomains [4]. Through the P2 subdomain, human noroviruses can bind histo-blood group antigens (HBGAs) [5,6], which serve as a host attachment factor for noroviruses and determine host susceptibility to norovirus infections [7–10]. Consequently, HBGA-based blockade ELISAs have been widely used as a surrogate neutralization assay to assess the protective potential of vaccine-elicited antisera or monoclonal antibodies (mAbs) [11].

On the basis of VP1 gene, human noroviruses can be classified into two major genogroups, genogroups I (GI) and II (GII), which are responsible for approximately 10% and 90% of norovirus infections, respectively [12]. GII can be further divided into at least 22 genotypes, among which GII.4 has been the dominant genotype and accounts for more than 70% of all norovirus outbreaks for the past two decades worldwide [13–18]. However, a GII.17 variant norovirus emerged in 2014–2015, causing a surging number of outbreaks in Asia, and later spread to other parts of the world [19–23]. These epidemiological data point to the possibility that GII.17 might soon replace GII.4 as the predominant cause of norovirus outbreaks on a global scale [24].

Phylogenetic analysis showed that GII.17 noroviruses can be sorted into three clusters: cluster I is defined by the prototype strain GII.17/C142/1978/GUF reported in 1978; cluster II strains emerged in 2005 [25,26]; and cluster III strains can be further divided into subclusters IIIa and IIIb with the latter emerging in the winter season of 2014–2015 [20,23,27–29]. X-ray structures of the P domains of three GII.17 strains representing clusters I, IIIa, and IIIb, respectively, showed a large number of surface-exposed substitutions from 2002 to 2014 and 2015, implicating a significant change in the herd immunity [30]. Recently, Qian et al. determined the structure of the P domain of a GII.17 cluster IIIb strain in complex with HBGAs, revealing the exact HBGA-binding site (HBS) on GII.17 capsid [31]. In a previous study, Lindesmith et al. compared the antigenicity of virus-like particles (VLPs) of GII.17 clusters I, II, and IIIb representative strains by an HBGA binding blockade assay and it was found that antisera against each of the VLPs exhibited different blockade antibody profiles [26], suggesting that the three GII.17 clusters are antigenically distinct at blockade epitopes. However, thus far, knowledge on the location of GII.17-specific blockade epitopes remains limited primarily due to the lack of GII.17-specific blockade mAbs that could allow for definitive epitope identification.

In the present study, we generated and characterized four mAbs (designated as M1 to M4) from mice immunized with VLP of a GII.17 cluster IIIb strain. Among the four mAbs, M1 and M3 reacted specifically with the cluster IIIb VLP and blocked its binding with HBGAs, indicating that M1 and M3 are GII.17 cluster IIIb-specific blockade mAbs. Epitope mapping revealed that M1 and M3 recognized the same highly exposed epitope consisting of residues 293–296 and 299 in the P2 subdomain.

## Materials and methods

### VLP production

Norovirus GI.1, GII.4, and GII.17 VLPs were produced in *Pichia pastoris* yeast. Norovirus strains selected for VLP production include GI.1 strain Norwalk (GenBank ID: M87661), GII.4 strain Hu/GII.4/Huzhou121/2012/CHN (GenBank ID: KC473544), GII.17 strain GII.17/142700/Shanghai/2014 (GenBank ID: KT380915; hereafter referred as GII.17-KT), GII.17 strain Hu/GII/JP/2014/GII.P17_GII.17/Kawasaki323 (GenBank ID: AB983218; hereafter referred as GII.17-AB), and GII.17 strain Hu/NoV/Katrina-17/2005/US (GenBank ID: DQ438972; hereafter referred as GII.17-DQ). GII.17 strain information is listed in Table 1. VP1 gene of each strain was codon-optimized, synthesized, and cloned into the backbone vector pPink-HC (Invitrogen, USA). The resulting plasmids were used to individually transform yeast PichiaPink^TM^ Strain 1 (Invitrogen) as described previously [32]. The resulting yeast transformants were screened for VLP expression by ELISA and the identified high-expressor clones were used for VLP production and purification. Briefly, the induced yeast cells were collected, resuspended in 0.15 M PBS buffer (pH ∼7.0) and lysed with an ultra-high pressure cell disrupter (JNBIO, China). The lysates were centrifuged and the resulting supernatants were precipitated in 0.2 M NaCl and 10% (W/V) PEG 8000 at 4°C. The precipitates were centrifuged, resuspended in 0.15 M PBS buffer and clarified by high-speed centrifugation. VLPs in the supernatants were pelleted by 20% sucrose cushion ultracentrifugation at 112,700 g for 4 h and resuspended in 0.15 M PBS buffer. The clarified supernatants were further purified by 10–50% sucrose gradient ultracentrifugation at 270,000*g* for 3 h. The fractions containing VLPs were pooled and concentrated by another round of sucrose cushion ultracentrifugation. At last, the VLPs were resuspended in 0.15 M PBS buffer, analysed by SDS-PAGE and ELISA as described below and quantified by Bradford assay.

**Table 1.** VLPs of different GII.17 clusters used in this study.

VLP	Strain	GenBank ID	Cluster
GII.17-KT^a^	Hu/GII.17/142700/Shanghai/2014	KT380915	IIIb
GII.17-AB	Hu/GII/JP/2014/GII.P17_GII.17/Kawasaki323	AB983218	IIIa
GII.17-DQ	Hu/NoV/Katrina-17/2005/US	DQ438972	II

^a^ GII.17-KT VLP was used as the immunogen for generation of the mAbs.

In addition, a number of constructs for expressing chimeric VLPs were created by replacing different portions of the VP1 of the GII.17-AB strain with the counterparts from the GII.17-KT strain. Protocols for producing chimeric VLPs were the same as those for producing parental VLPs.

### Mouse immunization and generation of hybridomas

For mouse immunization, VLPs of strain GII.17-KT were expressed in *Pichia pastoris* yeast as described above. The mouse immunization study was approved by the Institutional Animal Care and Use Committee at the Institut Pasteur of Shanghai and the animals were cared for in accordance with the institutional guidelines. Female Balb/c mice were immunized with 5 µg of the GII.17 VLP plus 500 µg of Alum adjuvant (Invivogen, USA) at weeks 0, 2, and 4. The mice were given a booster immunization with 15 µg of VLP at week 7. Three days later, splenocytes were isolated from the immunized mice and then fused with SP2/0 myeloma. The resulting hybridomas were screened for production of VLP-binding antibodies by ELISA as described below. MAbs were purified from positive hybridoma clones using HiTrap^TM^ Protein G affinity column (GE Healthcare, USA). The isotypes of the MAbs were determined by ELISA using an SBA Clonotyping System-HRP Kit (SouthernBiotech, USA). A norovirus cross-reactive mAb, 7D8, was prepared from a mouse immunized with the insect cells-derived GII.4 VLP [33] according to the protocol described above.

### VLP-binding ELISA

To determine the VLP-binding property of the mAbs, 96-well ELISA plates were coated with 50 ng/well of purified VLPs in PBS (pH ∼7.4) and incubated overnight at 4°C. Plates were blocked using 5% non-fat milk diluted in PBST for 1 h at 37°C. Then, the plates were incubated with each of the mAbs (50 ng/well) diluted in PBST containing 1% non-fat milk for 2 h at 37°C, followed by incubation with an horseradish peroxidase (HRP)-conjugated secondary antibody for 1 h at 37°C. After colour development, the absorbance was determined at 450 nm using a 96-well plate reader. In some cases, 100 µl/well of VLP-containing yeast lysates were used for coating in ELISA. For these samples, their reactivity with M1, M2, or M3 was normalized against their reactivity with the broadly reactive mAb M4, and calculated as: Relative binding = (OD450 detected with M1/M2/M3 – OD450 value of blank)/(OD450 detected with M4 – OD450 value of blank).

### Biolayer interferometry assay

Binding affinity of mAbs to GII.17 VLP was analysed by biolayer interferometry. Briefly, VLP was labelled with biotin using an EZ-Link sulfo-NHS-LC-LC-biotin kit (Thermo Scientific). The streptavidin (SA) biosensor tips were dipped into VLP-biotin solution for nearly 8 min. Following a rinse in kinetics buffer, the VLP-immobilized biosensor tips were allowed to associate with antibody at different concentrations and then dissociate in kinetics buffer. The VLP-bound biosensor was also allowed to associate with kinetics buffer alone (without antibody) to serve as a loading control. Data were processed using Octet data analysis (v11.0) software (ForteBio).

### VLP-mucin binding blockade assay

96-well plates were coated with 500 ng/well of pig gastric mucin (PGM) Type III (Shanghai Yuanmu Biotech, China) in PBS (pH ∼7.4), incubated for 4 h at room temperature, and then blocked with 5% no-fat milk in PBST at 4°C overnight. Serially diluted mAb samples (50 µl) was mixed with an equal volume of VLP (0.5 µg/ml), and incubated for 1 h at room temperature. Then, the VLP/mAb mixtures were added to the PGM-coated plates and incubated for 1 h at room temperature. After three washes, the plates were incubated with a rabbit anti-GII.17 polyclonal antibody for 1 h at room temperature, followed by incubation with HRP-conjugated anti-rabbit IgG (sigma) for 1 h at room temperature. After colour development, the absorbance was determined at 450 nm in a 96-well plate reader. The 50% inhibition concentration (IC50) was defined as the lowest mAb concentration that blocked at least 50% of VLP binding compared to levels determined in the absence of antibody pretreatment.

### VLP attachment inhibition assay

Blockade of VLP attachment to the 293T-FUT2 cells by the mAbs was determined as described previously with some modifications [34]. Briefly, 293T-FUT2 cells were seeded in poly-L-lysine-coated 96-well culture plates and incubated overnight. The cells were fixed with 4% paraformaldehyde for 30 min and washed with PBS followed by blocking. 50 μl/well of serially diluted mAb samples were incubated with equal volume of VLP (1 μg/ml) at 37°C for 1 h, and the mixtures were then added onto the 293T-FUT2 cells. After incubation at 4°C for 1 h, the cells were washed with PBS and then incubated with a polyclonal antibody against GII.17 VLP followed by an HRP-conjugated secondary antibody. After colour development, absorbance at 450 nm was measured. Anti-SARS-COV-2 mAb 2H2 [35] was used as isotype control in this assay. IC50 was defined as the lowest mAb concentration that blocked at least 50% of VLP attachment compared to levels determined in the absence of antibody pretreatment.

### Sequence alignment

VP1 amino acid sequences from the representative norovirus strains were aligned using CLC Sequence Viewer software (v6.8).

### Structural modelling

The locations of the M1/M3 epitope were shown on the P domain dimer X-ray crystal structure (PDB: 5F4O) of GII.17 norovirus isolate Kawasaki308 (GenBank ID: LC037415) using Chimera software (v1.11.2). The HBGA binding pocket residues were identified according to the previously reported crystal structures of P domain of GII.17-2014/15 complexed with A-trisaccharide (PDB: 5ZV5) or B-trisaccharide (PDB: 5ZV7) [31].

## Results

### Generation and binding characteristics of the anti-GII.17 mAbs

Splenocytes from mice immunized with GII.17 VLP (strain GII.17-KT; see Table 1) were fused with SP2/0 myeloma cells to generate hybridomas. Culture supernatants from the resulting hybridomas were screened by ELISA for their reactivity to GII.17 VLP. Four individual hybridoma clones, designated M1 to M4, respectively, were found to be ELISA-positive (Table 2). Isotyping assay showed that all of these four clones are IgG1 antibodies (Table 2).

**Table 2.** Characteristics of the anti-GII.17 mAbs.

MAb	Hybridoma ID	Isotype	Binding activity to GII.17 VLP^a^	Binding affinity with GII.17 VLP^b^		
				KD (nM)	Kon (1/Ms)	Kdis (1/s)
M1	1D3	IgG1	+++	<0.001	2.9 × 10^5^	<1.0 × 10^−7^
M2	2C1	IgG1	+++	<0.001	5.82 × 10^4^	<1.0 × 10^−7^
M3	3A3	IgG1	+++	0.01	4.84 × 10^5^	5.57 × 10^−6^
M4	4B1	IgG1	+++	0.39	1.88 × 10^5^	5.99 × 10^−5^

^a^ 50 μl supernatants from hybridoma cultures were used for analysis by ELISA. +, OD450 > 0.15; ++, OD450 > 0.3; +++, OD450 > 0.5.

^b^ KD (equilibrium), Kon and Kids of the purified mAbs towards GII.17 VLP was determined by BLI.

**Figure 1.**
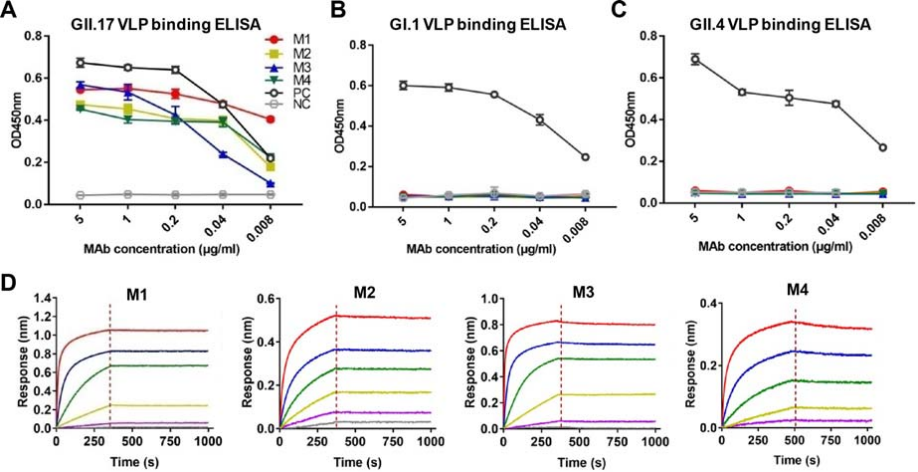
Binding properties of the anti-GII.17 mAbs. (A) Reactivity of the mAbs with GII.17-KT VLP in ELISA. (B) Reactivity of the mAbs with GI.1 VLP in ELISA. (C) Reactivity of the mAbs with GII.4 VLP in ELISA. Data are mean ± SD of triplicate wells. PC, a norovirus cross-reactive antibody 7D8 serving as a positive control. NC, anti-SARS-COV-2 mAb 2H2 serving as a negative control. (D) Binding affinities of the M1 to M4 mAbs to the GII.17-KT VLP determined by biolayer interferometry analysis. Association and dissociation steps are divided by dashed red lines.

To evaluate the binding specificity of the four mAbs, we performed ELISA with GI.1 VLP, GII.4 VLP, or GII.17 VLP as coating antigen. All of the four anti-GII.17 mAbs were found to react with GII.17 VLP in an antibody dose-dependent manner (Figure 1(A)), whereas none of them showed binding activity to GI.1 VLP or GII.4 VLP regardless of antibody doses (Figure 1(B,C)). In contrast, a broadly reactive mAb, 7D8, bound with each of the three VLPs (Figure 1(A–C)), validating the assays. These results demonstrate that the M1 to M4 mAbs can specifically distinguish GII.17 from the other norovirus types tested (GI.1 and GII.4).

Next, we performed biolayer interferometry assays to determine the binding affinity of the mAbs to GII.17 VLP. The results showed that the four mAbs had a high affinity to the VLP with KD values of <0.001 nM, <0.001, 0.01, and 0.39 nM for M1, M2, M3, and M4 antibodies, respectively (Figure 1(D) and Table 2).

We then determine whether the MAbs recognized denatured GII.17 VLP in western blot assays. It was found that none of the four mAbs produced positive signal whereas the rabbit anti-GII.17 VLP antisera yielded a ∼55KD band representing the denatured VP1 protein (data not shown), suggesting that the four anti-GII.17 mAbs only recognize conformational epitopes.

### Blockade activity of the four anti-GII.17 mAbs

**Figure 2.**
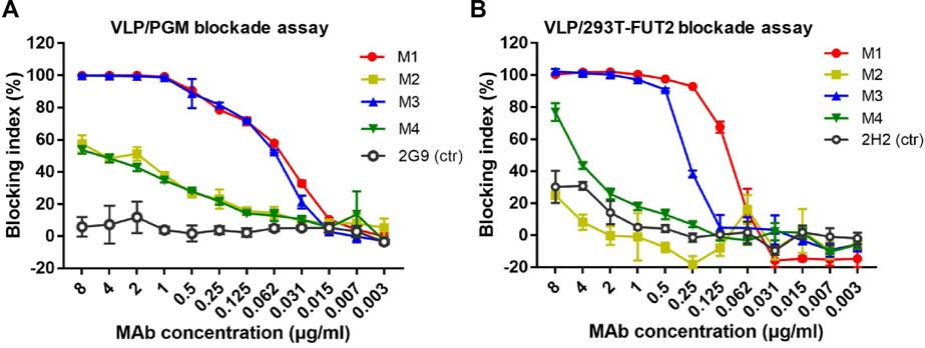
Blockade of the interaction between GII.17-KT VLP and HBGA by the anti-GII.17 mAbs. (A) The capacity of the mAbs to block GII.17-KT VLP interaction with HBGA-containing PGM III. Data are mean ± SD of triplicate wells. Anti-HBsAg mAb 2G9 served as the negative control (ctr) in the assay. (B) The capacity of the mAbs to block GII.17-KT VLP attachment to the HBGA-expressing 293T-FUT2 cells. Data are mean ± SD of triplicate wells. Anti-SARS-CoV-2 mAb 2H2 served as the negative control in the assay. Blocking index (%) was calculated for each sample by comparing the amount of VLP bound in the presence of antibody pretreatment to the amount of VLP bound in the absence of antibody pretreatment. Data are mean ± SD of triplicate wells.

The ability of the mAbs to block VLP interaction with histo-blood group antigens (HBGAs), a binding receptor for NoVs [36,37], was assessed by a well-established blockade assay, in which pig gastric mucin (PGM) type III was used as the source of HBGAs [38]. We found that M1 and M3 mAbs efficiently blocked GII.17 VLP binding to PGM in a dose-dependent manner with IC50s being 0.050 and 0.059 µg/ml, respectively, whereas M2 and M4 exhibited very low blockade activities (Figure 2(A)). The mAbs were also evaluated for their blockade activities in a surrogate neutralization assay based on the human α1,2-fucosyltransferase 2-transgenic 293T (293T-FUT2) cell line that expresses HBGAs on the surface [34]. It was found that M1 and M3 dose-dependently blocked GII.17 attachment to the 293T-FUT2 cells with IC50s of 0.09 and 0.27 µg/ml, respectively (Figure 2(B)), in agreement with the results from the PGM-binding blockade assays (Figure 2(A)). Note that for the cell-based blockade assay, there was no significant difference between the M1 and M3 groups (*p* > 0.05). Together, the above data demonstrate that M1 and M3 are potent blockade antibodies.

### Cross-reactivity of the mAbs towards different GII.17 clusters

**Figure 3.**
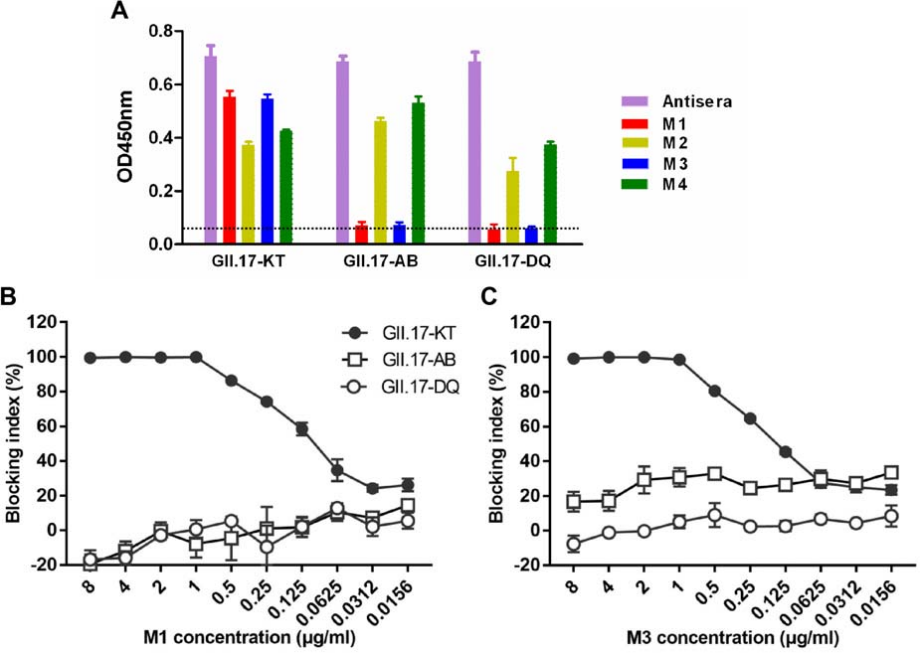
Cross binding and blocking activities of the MAbs towards VLPs representing different GII.17 clusters. (A) Binding of the mAbs to three GII.17 VLPs in ELISA. Anti-GII.17 sera served as a positive control in the assay. Data are mean ± SD of triplicate wells. Dotted line indicates the OD450nm value of the blank (without VLP coating). (B) Cross-blockade activity of the M1 mAb towards three GII.17 VLPs. (C) Cross-blockade activity of the M3 mAb towards three GII.17 VLPs.

The VLP strain GII.17-KT used to generate the four mAbs is a GII.17 cluster IIIb strain (Table 1). To assess the mAbs’ cross reactivity, VLPs of the strains GII.17-DQ and GII.17-AB, which belong to cluster II and cluster IIIa (Table 1), respectively, were produced and used as coating antigens in ELISA. As shown in Figure 3(A), the rabbit antisera raised against GII.17-KT VLP almost equally reacted with each of the three VLPs tested, serving as a control for antigen coating; M2 and M4 mAbs efficiently bound each of the three VLPs in the panel albeit there were slight variations in ELISA reactivity; in contrast, M1 and M3 mAbs did not show any significant reactivity to GII.17-AB and GII.17-DQ VLPs. Consistently, we found that M1 and M3 did not exhibit dose-dependent blockade effect on GII.17-AB and GII.17-DQ VLPs (Figure 3(B,C)). These data indicate that M2 and M4 antibodies bind common epitopes on the three VLPs whereas the epitopes recognized by blockade antibodies M1 and M3 are only present in GII.17-KT VLP.

### Mapping of the M1 and M3 antibody epitopes

**Figure 4.**
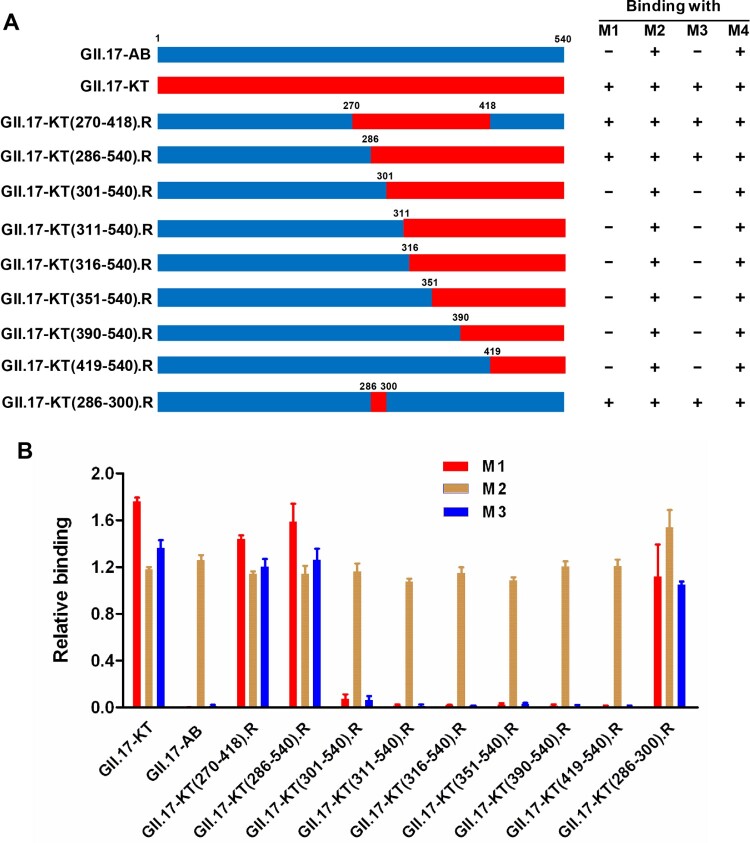
Epitope mapping for M1 and M3 mAbs. (A) Schematic diagram of the parental and chimeric VLP constructs and summary of the binding ELISA results. (B) Binding activities of the indicated parental and chimeric VLPs with M1, M2, M3, and M4 mAbs in ELISA. For a given VLP sample, its reactivity with M1, M2, or M3 was normalized against that with the broadly reactive mAb M4, and calculated as: Relative binding = (OD450 detected with M1/M2/M3 – OD450 value of blank)/(OD450 detected with M4 – OD450 value of blank). Data are mean ± SEM of triplicate samples.

To roughly locate the epitopes of blockade antibodies M1 and M3, we constructed a series of chimeric VLPs by exchanging fragments of the VP1 protein of GII.17-AB with the counterparts of GII.17-KT (Figure 4(A)). The resulting chimeric VLPs were assessed for their reactivity with the M1, M2, M3, and M4 mAbs in ELISA. The results were summarized in Figure 4(A) and representative data were shown in Figure 4(B). It was found that all of the chimeric VLPs could react strongly with the M2 and M4 antibodies, confirming their successful expression and assembly. Among these chimeric VLPs, only GII.17-KT(270-418).R which contained the exact P2 domain of the GII.17-KT VP1 protein, and GII.17-KT(286-540).R, could react with M1 and M3 antibodies, whereas the chimeric constructs with exchanges starting from the residue 301 or beyond failed to do so (Figure 4(B)). These data suggest that the binding epitopes of M1 and M3 reside within residues 286–300.

We further constructed an additional chimeric VLP by replacing the residues 286–300 in GII.17-AB with the corresponding ones from GII.17-KT (Figure 4(A)). The resulting chimeric VLP, GII.17-KT(286-300).R, was found to react not only with M2 and M4 but also with M1 and M3 antibodies (Figure 4(B)). This result shows that M1 and M3 antibodies recognize the same or overlapping epitopes located within residues 286–300 of GII.17-KT.

### Sequence alignment of the M1/M3 blockade epitope

**Figure 5.**
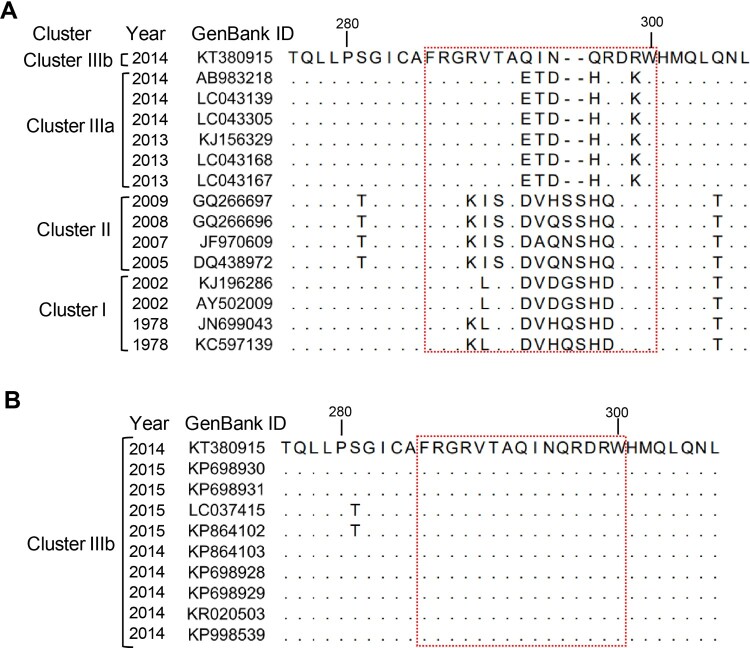
Alignment of the corresponding M1/M3 epitope sequences from (A) representative strains of different GII.17 clusters, or from (B) different GII.17 cluster IIIb strains. Information for each strain such as isolation date (year) and GenBank ID are shown. The M1/M3 epitope region (residues 286–300) was boxed with a red dash line. Dots represent residues identical to those of GII.17-KT (GenBank ID: KT380915).

Sequence alignment revealed that the sequences corresponding to the M1/M3 epitope are highly variable among GII.17-KT, cluster I, cluster II, and cluster IIIa strains (Figure 5(A)). In particular, the epitope sequence of GII.17-KT differed from the counterparts in GII.17-AB (cluster IIIa) by five amino acids, indicating these five residues (namely 293Q, 294I, 295N, 296Q, and 299R) are critical components of the M1/M3 epitope. Compared with GII.17-KT, GII.17-DQ has eight residues changed and two additional amino acids inserted in this region. We also compared the VP1 sequences of multiple cluster IIIb strains with that of GII.17-KT and found that they are identical in the M1/M3 epitope region (Figure 5(B)), indicating the epitope is conserved within cluster IIIb.

### Structural modelling of the M1/M3 blockade epitope

**Figure 6.**
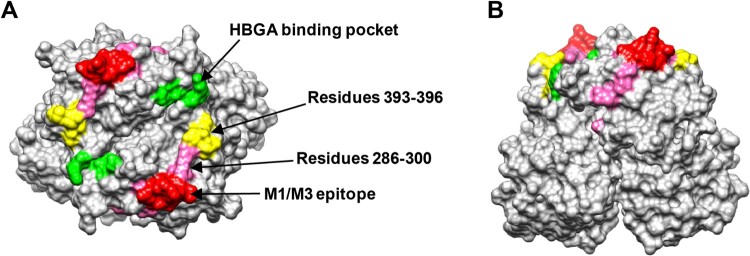
The M1/M3 epitope is surface exposed. (A and B) Model of the GII.17 P domain dimer (PDB: 5F4O) showing the M1/M3 epitope. (A) Top view. (B) Side view. The M1/M3 epitope (residues 293, 294, 295, 296, and 299) is shown in red and the other residues within 286–300 are shown in pink. The HBGA binding pocket is shown in green, and the previously proposed blockade epitope (residues 393–396) is shown in yellow.

Structural modelling shows that the M1/M3 epitope defined by residues 293–296 and 299 is highly exposed, forming a finger-like protrusion (Figure 6). This epitope has no overlap with a previously suggested GII.17 blockade epitope comprising residues 393–396 [26] while it is close to the HBGA binding site [31].

## Discussion

Prior to this study, information on GII.17 blockade epitopes is limited due mainly to the lack of GII.17-specific mAbs. A previous study, which combined bioinformatics analysis and measurement of polyclonal antisera’ blockade activity towards a small panel of time-ordered GII.17 VLPs, suggested that residues 393–396 within the GII.17 VP1 protein might form a varying blockade epitope [26]. However, exchange of residues 393–396 from the emergent cluster IIIb strain GII.17.2015 into the prototype strain GII.17.1978 (cluster I) resulted in decreased antibody reactivity and loss of HBGA binding [26], making the role of these residues as a blockade epitope unclear. In the present study, we showed that M1 and M3 mAbs could efficiently block VLP binding to HBGA-containing PGM and to HBGA-expressing 293-FUT2 cells (Figure 2). The binding sites for M1 and M3 were mapped to a region comprised of residues 286–300 of the VP1 protein of the cluster IIIb strain GII.17-KT (Figure 4). Sequence alignment showed that the region of residues 286–300 in GII.17-KT differs from that in GII.17-AB, a cluster IIIa strain with which M1 and M3 did not bind, by five amino acids, namely 293Q, 294I, 295N, 296Q, and 299R (Figure 5(A)). We further demonstrated that exchange of these five residues from GII.17-KT into GII.17-AB restored M1/M3 binding (Figure 4(B)). Collectively, our data convincingly show that residues 293Q, 294I, 295N, 296Q, and 299R of VP1 are involved in the GII.17 cluster IIIb-specific blockade epitope. Because the M1/M3 epitope is a conformational epitope, it cannot be ruled out the possibility that other residues may also play critical roles in the recognition of GII.17-KT VLP by the M1 and M3 mAbs. Further analysis (e.g. structure determination of the VLP/mAb complex) will be needed to clarify this issue. To our knowledge, this is the first study to experimentally map a blockade epitope in GII.17 noroviruses.

Structural modelling shows that the M1/M3 epitope forms a surface loop and is highly exposed (Figure 6) and is therefore easily targeted by the host immune system for the generation of neutralizing antibodies. The location of the M1/M3 epitope is similar to that of the previously reported evolving epitope A (residues 294-298, 368, and 372-373) in GII.4 strains [39–41]. Comparison of the corresponding epitope sequences from different GII.17 clusters reveal high variations in the M1/M3 epitope region (Figure 5(A)). Our work thus provides direct evidence supporting the notion that GII.17 is capable of antigenic drift, allowing for escape from herd immunity and increased circulation of the virus [26]. Additionally, our data also suggest viral evolution at the region encompassing residues 286–300 (which contains the M1/M3 epitope) may have contributed, at least in part, to the emergence of clusters IIIa and IIIb strains.

A previous study shows that HBGA binding of GII.17 cluster IIIb VLP could be blocked by antisera from mice immunized with cluster IIIb VLP but not by antisera against cluster I or II VLPs, indicating a lack of cross-cluster blockade activity for the antisera [26]. Note that cluster IIIa VLP was not produced and tested in that study [26]. Consistently, in the present study, we found that the blockade mAbs (M1 and M3) targeting the cluster IIIb VLP did not exhibit binding and blockade activities towards cluster II and cluster IIIa VLPs (Figure 3(B,C)). Together, these data suggest that the M1/M3 epitope is possibly a dominant antibody epitope determining the ability of GII.17 VLPs to induce blockade antibodies.

In summary, the present study generated a panel of anti-GII.17 mAbs and defined for the first time an evolving blockade epitope on the GII.17 norovirus capsid protein, providing insights into the evolution and persistence of GII.17 in humans and also information critical for future norovirus vaccine development.
